# Selective delivery of interleukine-1 receptor antagonist to inflamed joint by albumin fusion

**DOI:** 10.1186/1472-6750-12-68

**Published:** 2012-09-25

**Authors:** Mengyuan Liu, Yi Huang, Lei Hu, Guoping Liu, Xueping Hu, Dongxu Liu, Xiaosong Yang

**Affiliations:** 1 Center for Infection and Immunity Research, School of Life Sciences, Hubei University, Youyi Road 368, Wuhan, 430062, China; 2 College of Animal Sciences, Yangtze University, Jingmi road 88-2, Jingzhou, 434025, China; 3 The Institute of Medicinal Technology, Peking Union Medical College and Chinese Academy of Medical Science, Beijing, 10050, China

**Keywords:** Human serum albumin, Interleukin-1 receptor antagonist, Fusion protein, Rheumatoid arthritis, Pharmacokinetics, Pharmacodynamic

## Abstract

**Background:**

Interleukin-1 receptor antagonist, a cytokine that is highly therapeutic to rheumatoid arthritis and several other inflammatory diseases, exhibits rapid blood clearance and poor retention time on the target in clinical application due to its small size and lack of specificity to target tissue. Albumin has been widely employed as macromolecular carrier for drug delivery purpose to extend the plasma half-life of therapeutic molecules and has been shown to selectively accumulate and to be metabolized in the inflamed joints of patients with rheumatoid arthritis. This suggests that genetic fusion of IL-1ra to albumin can probably overcome the drawbacks of *in vivo* application of IL-1ra.

**Result:**

A recombinant protein, engineered by fusing human serum albumin (HSA) to the carboxyl terminal of IL-1ra, was produced in *Pichia pastoris* and purified to homogeneity. The fusion protein retained the antagonist activity of IL-1ra and had a plasma half-life of approximately 30-fold more than that of IL-1ra in healthy mice. *In vivo* bio-distribution studies demonstrated that the fusion protein selectively accumulated in arthritic paws for a long period of time in mice with collagen-induced arthritis, showing low uptake rates in normal organs such as liver, kidney, spleen and lung in contrast to IL-1ra alone. Moreover, this fusion protein was able to significantly improve the therapeutic efficacy of IL-1ra in collagen-induced arthritis mouse model.

**Conclusions:**

The fusion protein described here, able to selectively deliver IL-1ra to inflamed tissue, could yield important contributions for the therapy of rheumatoid arthritis and other inflammatory diseases.

## Background

Rheumatoid arthritis (RA) is a chronic inflammatory disorder characterized by systemic autoimmune attacking synovial joints, leading to articular destruction and functional disability and a wide array of extra-articular complications [1]. There is increasing evidence that Interleukin-1 (IL-1) plays an important role in several chronic inflammatory diseases, including RA [2]. The IL-1β-NF-κB axis is a key pathway in the pathogenesis of RA. NF-κB activation by IL-1β induces a widely spectrum of pro-inflammatory mediators that contribute to the inflammation in the synovium [2]. IL-1 also activates the fibroblast-like synoviocytes through the IL-1β-NF-κB pathway to produce a family of MMPs, resulting in collagen degradation and bone erosion [3,4]. Therefore, blocking the effect of an excess of IL-1 may provide a therapeutic option of RA. Interleukin-1 receptor antagonist (IL-1ra), a member of IL-1 family, is a naturally occurring cytokine that blocks biological activity of IL-1 through binding to IL-1 type I receptor with the same affinity as that of IL-1β. Recombinant human IL-1ra (rhIL-1ra), Anakinra, has been approved for the treatment of patients with moderate-severe rheumatoid arthritis (RA) and has been shown to slow cartilage degradation and to provide relief from joint symptoms when administered subcutaneously [5-7]. rhIL-1ra is also documented to have therapeutic potential for several other autoimmunity diseases associated with IL-1 dysregulation [8]. However, IL-1ra exhibits rapid blood clearance and poor retention time on the target in clinical application, due to its small size and lack of specificity to the target tissue [9]. High dosages and repeated injections are therefore required, which greatly influences patients' physical, psychological and economical situation. Additionally, systemic administration of a high dosage (150 mg/day) usually exposes patients to complications that include injection site reactions, serious bacterial infection, high risk of tumorigenesis, and neutropenia [6,10-14]. The pharmacokinetic properties of small protein drugs can probably be improved by covalent coupling to a suitable carrier, which should be characterized by such features as long circulation half-life, high accumulation in the target tissue, and low uptake rates in normal tissue, as well as low toxicity and general availability. The plasma protein albumin satisfies these requirements for drug delivery as demonstrated by a number of studies.

Albumin is the most abundant plasma protein with a molecular weight of 66.5 kDa. It is produced as a monomeric protein in the liver and has an average circulation half-life of 19 days in humans. This long serum half-life is due to a recycling process mediated by the neonatal Fc receptor (FcRn), similar to that observed for IgG molecules [15,16]. Albumin has a simple molecular structure and is highly stable. In addition to its role in regulating the osmotic pressure of plasma, albumin serves as a carrier for the transport of metabolites like long chain fatty acids, bilirubin, steroid hormones, tryptophan, and calcium. Albumin also binds with high affinity to a broad range of drugs influencing their pharmacokinetic properties [17]. Taking these advantages into consideration, albumin has been employed as macromolecular carrier for drug delivery purpose to improve pharmacokinetic properties and efficacy of therapeutic molecules [18-21]. In addition, albumin has been shown to selectively accumulate in the inflamed joints of RA patients and of mice suffering from collagen-induced arthritis [22-26], indicating it is an attractive carrier for targeted delivery of drugs to the inflamed sites. The antirheumatic conpound methotrexate covalently linked to albumin has shown promising activity in the collagen-induced arthritis model [26]. Non-covalently binding protein drugs to albumin by fusing these drugs to anti-albumin antibody fragments also results in prolonged half-lives and selective accumulation of the drugs in inflamed joints [27,28]. However, it has not been documented so far whether direct fusion of protein drugs to albumin has the same effect as mentioned above.

Herein, we report the engineering and production of a fusion protein that consists of human IL-1ra and HSA and its application prospect for RA therapy. We demonstrated that the fusion protein retained the bioactivities of IL-1ra and had a much longer serum half-life than IL-1ra in mice. The fusion protein also selectively accumulated in arthritic joints of mice for a long period of time, with low distribution rates in other organs such as liver, kidney, spleen and lung, in contrast to IL-1ra alone. In addition, the fusion protein exhibited a more pronounced therapeutic efficacy in mice arthritis model compared with IL-1ra. The findings reported herein indicate that the fusion protein is likely to be an ideal biological agent in the treatment of rheumatoid arthritis, and that genetic fusion of small protein drugs to albumin is a promising drug delivery approach for RA targeted therapy.

## Results

### Engineering and identification of fusion protein

**Figure 1:**
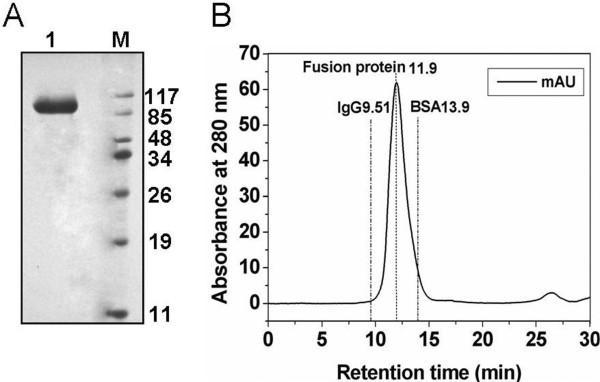
**Purification and gel filtration analysis of the fusion protein.** (**A**) SDS/PAGE under reducing condition shows the result of purification. Lane M, the molecular mass standards(kDa); lane 1, the fusion protein. The molecular masses in kDa are given on the right of the gel. (**B**) Gel filtration analysis of the fusion protein on a TSK-G3000-SW_XL_ column. The molecular mass standards are IgG (150 kDa) and BSA (66 kDa).

The fusion gene, inserted into the plasmid vector pHBM905B, was confirmed by DNA sequencing, and the positive transformants (*P*. pastoris GS115 (his4)) were identified by specific amplification of the fusion gene (PCR) using genomic DNA as templates. The results demonstrated that the target fragment of the vector pHBM905B/IL-1ra-HSA was integrated into the genome of *P*. pastoris GS115 (his4). The fusion gene was inserted into the downstream of α-factor signal peptide within the same frame and under the control of AOX1 (Alochol Oxidase) promoter, allowing the fusion protein to be expressed and successfully secreted into the culture medium. The fusion protein was expressed as a 91 kDa functional protein in the culture medium and the expression level was positively correlated with the induction time. The highest expression level was achieved after 120 h of induction, which accounted for approx. 60% of the total protein in the medium. The target protein was further identified by immunoblotting. After ammonium sulfate precipitation, the fusion protein was routinely purified to greater than 90% purity using IMAC (Figure 1). We routinely obtained 18–20 mg of fusion protein from a 1 liter medium supernatant. On concentration by ultrafiltration, a concentration of at least 1 mg/ml for fusion protein could be obtained. The purified protein was further checked by gel filtration chromatography. The fusion protein was eluted at a retention time of 11.9 min, which corresponded to a molecular mass of about 91 kDa consistent with a monomer (Figure 1). No polymer peaks and other protein peaks were observed, which indicated that the fusion protein was homogeneous in the form of monomer with a high purity.

### In vitro bioactivity of fusion protein

**Figure 2:**
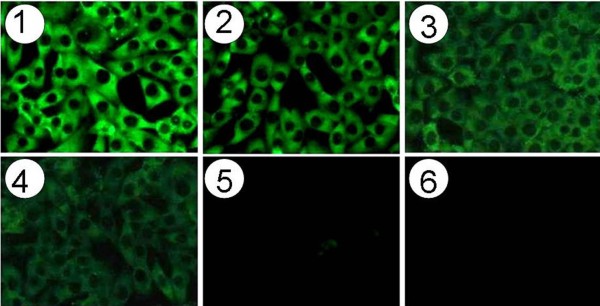
**Immuno-fluorescence staining detection for the receptor-binding of the fusion protein.** A375.S2 cells incubated with 100 μg/ml fusion protein (**1**), with 10 μg/ml fusion protein (**2**), with 1 μg/ml fusion protein (**3**), with 0.1 μg/ml fusion protein (**4**), with 0 μg/ml fusion protein (**5**), and with 100 μg/ml HSA (**6**). FITC-labeled anti-His tag mAb is used in this assay. The fusion protein, IL-1ra and HSA have a His tag at the C-terminal.

**Figure 3:**
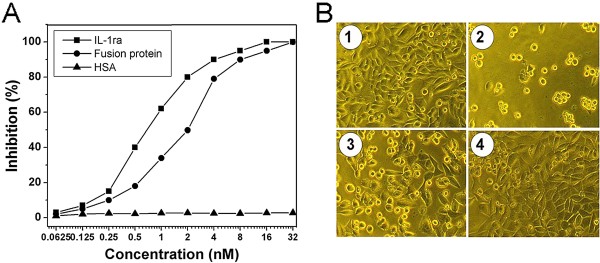
**Dose-dependent inhibition of the cytotoxic effect of IL-1β to A375.S2 cells.** (**A**)% levels of inhibition under varied inputs of the fusion protein, IL-1ra, and HSA. (**B**) Inhibition of IL-1β-induced cytotoxicity to A375.S2 cells as shown by photomicroscopy. Cells were incubated in the presence of 32 nM fusion protein (**1**), 1 ng/ml (0.06nM) IL-1β alone (**2**), 0.06nM IL-1β plus 2 nM fusion protein (**3**), and 0.06nM IL-1β plus 32 nM fusion protein (**4**). There is no difference: between (1) and cells with medium alone (not shown); between (2) and the cells in 0.06nM IL-1β plus 32 nM HSA (not shown).

IL-1ra is a natural antagonist that blocks IL-1 action by competitively binding to the IL-1 receptors, thus preventing the biological response mediated by IL-1. Immunofluorescence analysis revealed a dose-dependent binding of fusion protein to IL-1 receptor positive A375.S2 cells. As expected, IL-1ra also bound to A375.S2 cells in a dose-dependent manner, whereas no binding of HSA to A375.S2 cells was observed (Figure 2). The experiments clearly demonstrate that the receptor binding ability of IL-1ra is not impaired by fusion to HSA. IL-1 is cytotoxic to A375.S2 cell through IL-1 receptor-mediated signal pathway, and the cytolytic activity of IL-1 can be blocked by IL-1ra. Therefore, the fusion protein was also examined for its capacity to inhibit IL-1-dependent A375.S2 cells killing. As shown in Figure 3, both IL-1ra and fusion protein inhibited the cytolytic activity of 1 ng/ml (0.06 nM) of IL-1β to A375.S2 cells in a dose-dependent manner. For the fusion protein, the 50% and the 100% level of inhibition were achieved at an input of approx. 2 nM and 32 nM respectively, showing a slight reduction of activity compared with IL-1ra. By contrast, HSA did not show any inhibitory effect. These results indicate that fusion protein retains IL-1 antagonist activity, similar to that of IL-1ra.

### Serum albumin levels in arthritic mice and healthy control mice

The serum albumin level in arthritic mice was 23.1 ± 3.1 g/L, which was significant lower than that in healthy mice (35.7 ± 3.8 g/L). On the contrary, the albumin level (1.07 ± 0.18 mg/g) in the inflamed joints of arthritic mice was about 6-fold more than that (0.18 ± 0.07 mg/g) in the joints of healthy mice. The results suggest that mice with CIA can also develop to hypoalbuminemia, a serious complication due to accelerated metabolism of albumin in inflamed joints as demonstrated by a number of studies in RA patients [22-25]. This is the theoretical basis of our albumin-based targeted therapy.

### Pharmacokinetics analysis of fusion protein

**Figure 4:**
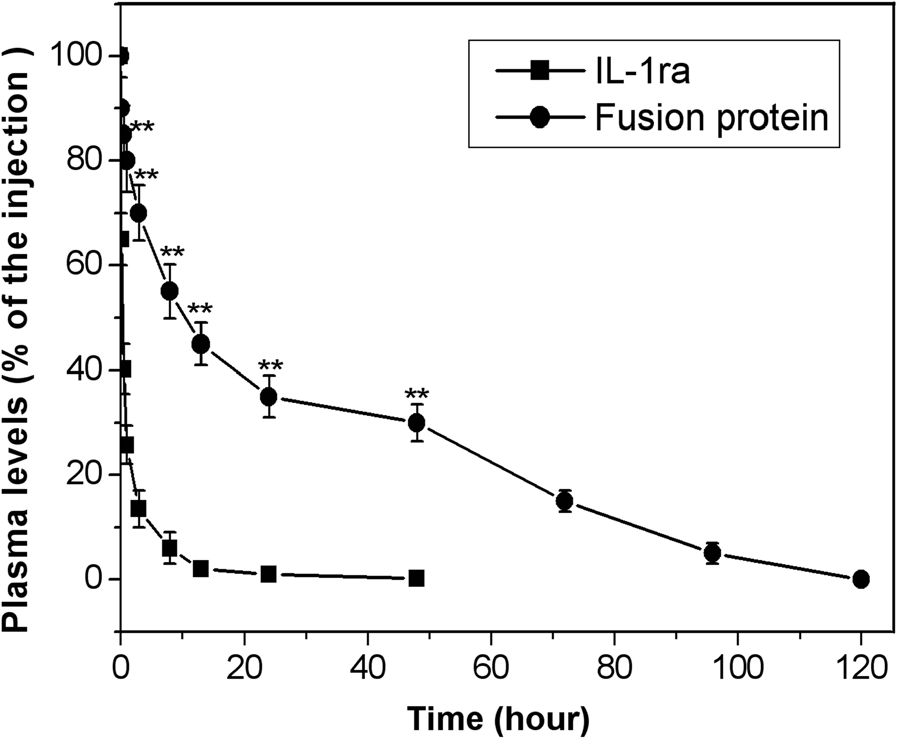
**Plasma levels of**^**131**^**I-labeled fusion protein and**^**131**^**I-labeled IL-1ra after injection to healthy mice though the tail vain.** Each data point represents mean ± SEM (n = 5). ** = p < 0.01 versus IL-1ra.

The amounts of radiaolabeled fusion protein and radiolabeled IL-1ra present in blood circulation in the healthy mice at different time points are shown in Figure 4. At 10 min post-injection, about 90% of the fusion protein tracer could be found in the circulation, compare with 65% of IL-1ra tracer. The circulation tracer of IL-1ra decreased to about 40% after 30 min, and was completely cleared from the circulation after 8 h. By comparison, the fusion protein decreased at a much lower rate than the levels of IL-1ra, with the circulation tracer of approx. 85% after 30 min and approx.55% after 8 h. At 48 h post-injection, approx.30% of the fusion protein could be still observed in the circulation. Generally, it would take 120 hours for the fusion protein to be completely cleared from the circulation. The data indicate that the fusion protein has a longer retention time in blood circulation compared with IL-1ra. Analysis of the obtained curves by the GraphPad software yielded the half-life of the fusion protein was 9.8 ± 1.7 h, compared to 0.34 ± 0.05 h for IL-1ra. The plasma levels of fusion protein in arthritic mice at different time points showed no significant difference in comparison with those in healthy mice, and so did the plasma levels of IL-1ra (not shown).

### Uptake in paws and biodistribution of fusion protein

**Figure 5:**
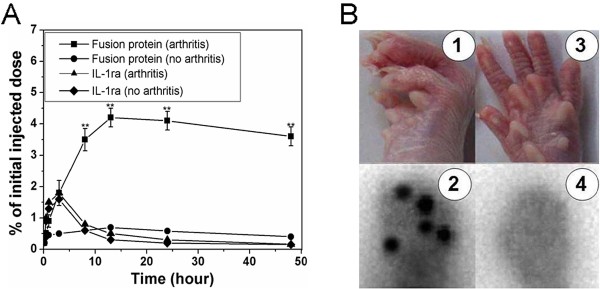
**(A) Uptake kinetics of**^**131**^**I-labeled fusion protein and**^**131**^**I-labeled IL-1ra in hind paws of mice with (n = 5) and without CIA (n = 5) after intravenous injection.** Data are expressed as mean ± SEM. ** = p < 0.01 versus uptake rates of ^131^I-labeled IL-1ra in hind paws of arthritic mice or healthy mice and uptake rates of ^131^I-labeled fusion protein in hind paws of healthy mice. (**B**) Autoradiogram for the selectively accumulation of the fusion protein in the inflamed joint of mice. (1) and (2), inflamed paw; (3) and (4), normal paw.

**Figure 6:**
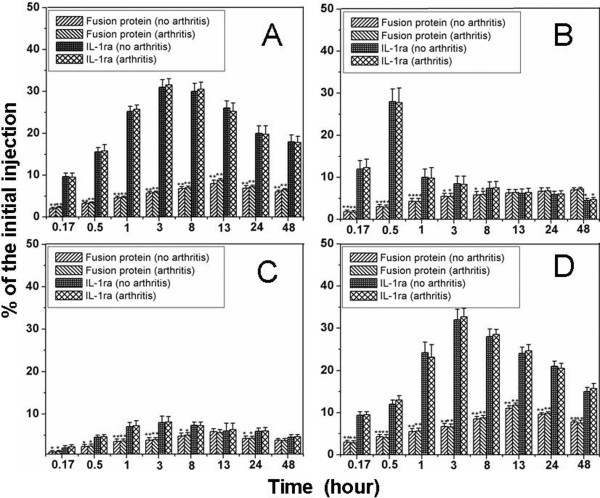
**The uptake kinetics of**^**131**^**I-labeled fusion protein and**^**131**^**I-labeled IL-1ra in the liver (A), kidney (B), spleen (C), and lung (D) of mice with (n = 5) and without CIA (n = 5).** Data are expressed as mean ± SEM. * = p < 0.05 and ** = p < 0.01 versus uptake rates of ^131^I-labeled IL-1ra in liver, kidney, spleen, and lung of arthritic mice or healthy mice.

On the 11th day after the second immunization, all the mice showed the first sign of disease, predominantly in the hind paws and hind ankles. The disease was progressive and achieved a clinical score of 12 to 16 in most of mice 30 days after the second immunization. On this day, the arthritic mice and healthy mice were assigned for the biodistribution analysis. The uptake of radiolabeled fusion protein and radiolabeled IL-1ra by healthy and inflamed hind paws is illustrated in Figure 5. The maximal accumulation of fusion protein in arthritic paws was achieved 13 h after injection. On average, an individual inflamed hind paw accumulated 4.2 ± 0.3% of the initial injected radioactivity after 13 h. This value exceeded that of healthy hind paw by approx. 6-fold. After 48 h, 3.6 ± 0.3% of the fusion protein was detectable in the inflamed hind paws, which was about 15-fold greater than that remaining in healthy hind paws. The autoradiograms showed the similar result. After 48 h, a selectively accumulation of radiolabeled fusion protein in the inflamed hind paws was observed, whereas accumulation of the fusion protein in healthy hind paws was not detectable (Figure 5). In contrast to the fusion protein, radiolabeled IL-1ra showed no accumulation in inflamed hind paws. Rapid elimination of radiolabeled IL-1ra was found in hind paws affected by arthritis. The maximal uptake rate of radiolabeled IL-1ra was obtained 3 h post-injection with the value of 1.8 ± 0.2%, decreasing to 0.8 ± 0.2% after 8 h and to 0.16 ± 0.02% after 48 h, 20-fold less than that of radiolabeled fusion protein at the same time point. No significant differences were found between the uptake rates of radiolabeled IL-1ra in inflamed hind paws and those of radiolabeled IL-1ra in healthy hind paws. The forepaws showed the similar results (not shown). The biodistribution of radiolabeled fusion protein and radiolabeled IL-1ra in liver, kidney, spleen and lung of arthritic mice and healthy mice at different time points is shown in Figure 6. The maximal uptake rates of fusion protein in liver, spleen and lung was achieved 13 h after injection, compared with 3 h for IL-1ra. The uptake rates of the fusion protein in these organs of arthritic mice showed no significant differences compared with those of the fusion protein in the corresponding organs of healthy mice, and IL-1ra uptake by these organs showed the similar results. However, the uptake rates of the fusion protein were significantly lower than those of IL-1ra in these organs, especially in the liver and lung, of both arthritic mice and healthy mice. The organ distribution pattern of the fusion protein was quite similar to that of HSA, i.e. the highest concentration was found in blood, followed by lung, liver, kidney and spleen. In the kidney, the maximal uptake of IL-1ra appeared 30 min after injection, with the amount as approx. 6 times as that of the fusion protein. It is generally thought that IL-1ra was rapidly eliminated from the blood via glomerular filtration in the kidney. These results demonstrate that fusion of HSA to IL-1ra significantly increased its tissue specificity and reduced its distribution to other organs.

### Inhibition of established arthritis in mice

**Figure 7:**
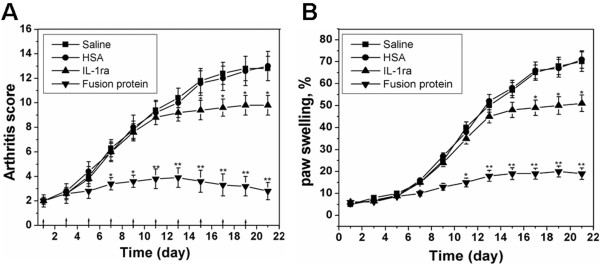
**The therapeutic efficacy of proteins in established arthritis in mice.** (**A**) Arthritis scores of CIA mice (n = 5) during 21 days treatment. (**B**) Increment in paw swelling of CIA mice (n = 5) during 21 days treatment. Day 1 corresponds to the first day that clinical arthritis was found. The upward arrows mean the day when the proteins were administrated. Data are expressed as mean ± SEM. * = p < 0.05 and ** = p < 0.01 versus the clinical score and paw swelling of arthritic mice treatment with saline.

Treatment of mice CIA was carried out on the 11th day after the second immunization, coinciding with the onset of the disease. The severity of arthritis was monitored by clinical score and paw swelling. Figure 7 shows the progression of arthritis during the treatment period. The arthritis score in the saline-treated group increased slightly and reached a mean value of 12.8 at the end of the treatment. The same variations were also observed in the paw swelling of the arthritic mice given saline. In comparison with the mice treated with saline, a pronounced reduction in clinical score and paw swelling was observed in mice treated with the fusion protein. IL-1ra treatment, however, showed a lower suppression of arthritis progression compared with the fusion protein. Significant reduction of clinical score and paw swelling only appeared at the end of the treatment. No significant therapeutic efficacy was found in the HSA-treated group.

### Histopathology

**Figure 8:**
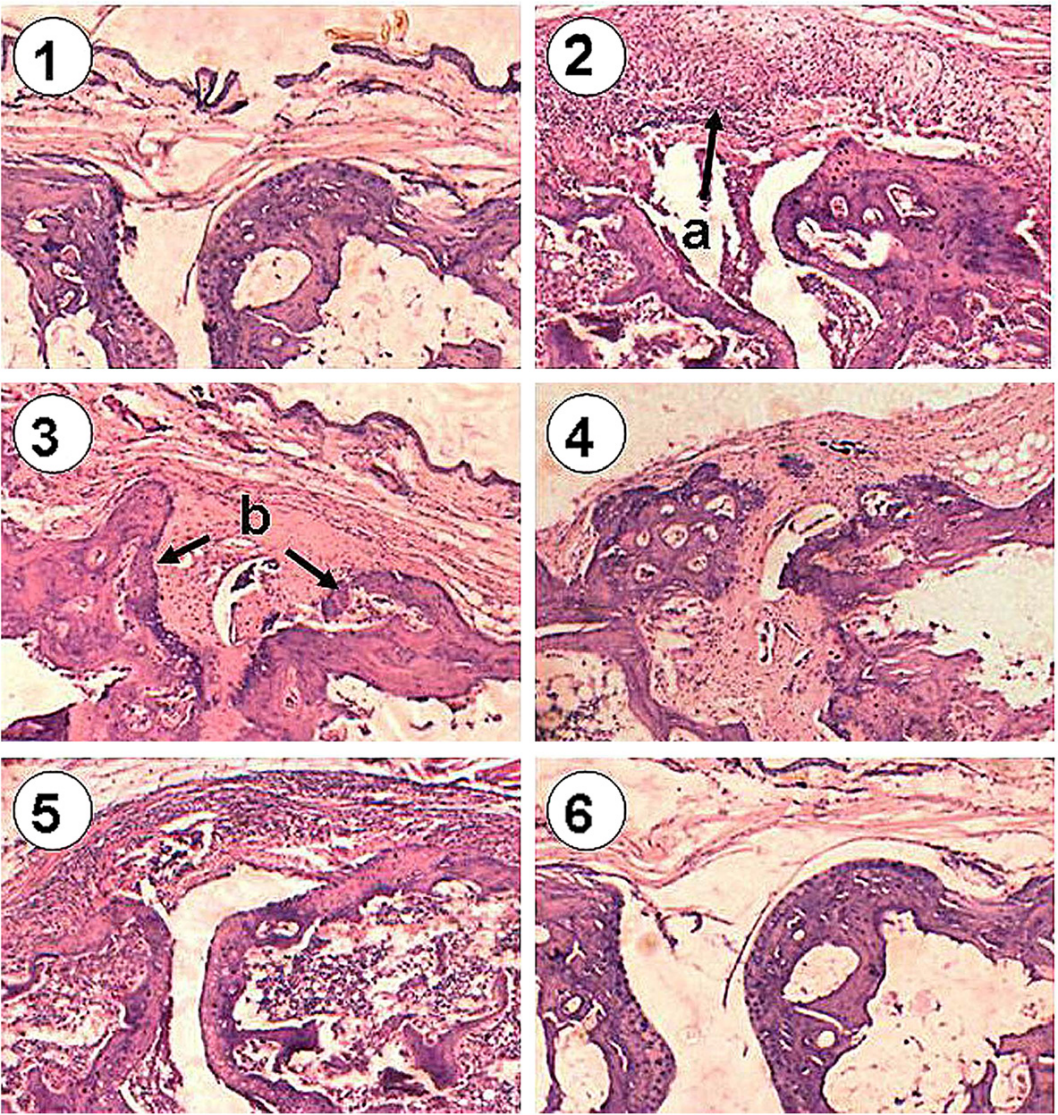
**Representative ankle joints histology in mice among the experimental groups (1), normal control; (2) and (3), CIA plus saline; (4), CIA plus HSA; (5) CIA plus IL-1ra; (6) CIA plus the fusion protein.** (a), synovial hyperplasia and exudation of inflammatory cells into the synovial space; (b), synovial hyperplasia, pannus formation, and erosion of bone and cartilage.

The hind limbs of mice from different groups were removed for histological analysis of ankle joints. The histological score given for the saline-treated group was 15.4 ± 2.1 at the end of the experiment. A significant reduction in histological score (10.5 ± 1.6) was observed in the IL-1ra-treated group (p < 0.05). By comparison, the fusion protein treatment resulted in a histological score of 3.2 ± 0.6, which is more significant (p < 0.01). As expected, no significant reduction in histological score (15.2 ± 2.5) could be found for HSA treatment. Representative ankle joint histopathology of the experimental groups is shown in Figure 8. Arthritis in mice with CIA is characterized by synovial hyperplasia, pannus formation, exudation of cells into the joint space, and erosion of bone and cartilage. A massive influx of inflammatory cells, synovial hyperplasia, pannus formation, and cartilage erosion in the joint space of CIA mice given saline was obviously observed compared with the normal control mice. The mice treated with HSA showed the same pattern of joint damage as that of arthritic mice. By comparison, mice treated with the fusion protein revealed pronounced reduction in inflammation and joint destruction to the extent that the synovial membrane in the joints was almost like the normal synovium, except for a mild synovial hyperplasia and other characteristics of inflammation. A less degree of arthritis severity was found in the mice treated with IL-1ra. Despite no visible damage to the joint, such as bone erosion and cartilage desquamation, the influx of inflammatory cells and synovial hyperplasia were somewhat pronounced.

### Cytokine expression

**Figure 9:**
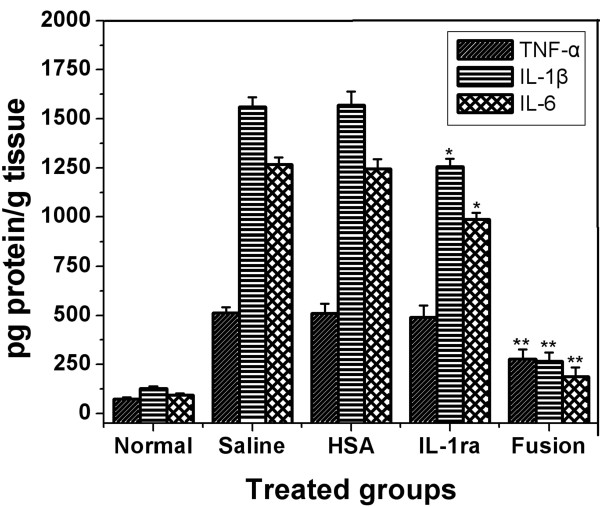
**Inflammatory cytokines expression in the joint of mice among the experimental groups.** Data are expressed as mean ± SEM. * = p < 0.05 and ** = p < 0.01 versus inflammatory cytokines expression in the joints of arthritic mice treated with saline.

To further confirm the anti-arthritis effect of the fusion protein, the expression of inflammatory cytokines such as TNF-α, IL-1β, and IL-6 in the joint of mice was analyzed by a quantitative ELISA at the end of treatment. As shown in Figure 9, in normal mice, the mean protein level for TNF-α, IL-1β, and IL-6 was approx. 70, 100, and 80 pg/g of tissue. A marked increase for the three cytokines was found in the joints of mice given saline. Especially for IL-1β, the amount was increased to approx. 15-fold more than that in the joints of normal mice. IL-1ra treatment gave a significant reduction in protein levels of IL-1β and IL-6, whereas no significant reduction was found for TNF-α. On the other hand, fusion protein treatment resulted in more pronounced reduction in the protein levels of the three cytokines. No downregulation of the three cytokines was observed with HSA treatment. The results, together with the above ones, suggest that fusion of HSA to IL-1ra markedly increases the therapeutic efficacy of IL-1ra to mice CIA, and that the HSA component of the fusion protein exhibits no therapeutic effect but exerts a synergistic effect of the fusion on the activity of IL-1ra.

## Discussion

The present study was carried out to improve the pharmacokinetic profiles of IL-1ra, a naturally occurring cytokine that is highly therapeutic to RA and several other inflammatory diseases. Recombinant DNA technology was used to fuse HSA to IL-1ra in order to enhance the clinical use of IL-1ra. The fusion protein was successfully expressed in yeast cells and secreted to the culture medium in functional form. A good yield was obtained followed by a purification procedure of IMAC, and the fusion protein was confirmed to be homogeneous in the form of monomer by gel chromatography analysis. In vitro functional studies showed that the fusion protein could bind to IL-1 receptor and inhibit the cytotoxic effect of IL-1β. This suggests that the antagonist activity of IL-1ra was clearly acquired by the fusion protein. However, the bioactivity of the fusion protein showed a slight reduction compared with IL-1ra. Macromolecularisation has been reported to cause a reduction of activity [29]. It is thought that fusion may impose steric hindrance between the substrate and the active site of the original functional protein. This drawback will be probably overcome by using different type of linkers and changing the arrangement of IL-1ra and HSA [29]. Further studies should be focused on how to improve the bioactivity of the fusion protein.

Two aspects support our concept of albumin-based targeted drug delivery. On the one hand, the generation of albumin fusions has proven to be an effective approach to improve the plasma half-life of small proteins, including hormones, cytokines, and antibody fragments [30-33]. On the other hand, several findings indicate that high amounts of albumin accumulate and are metabolized in inflamed joints of RA patients. It is well known that patients with active RA frequently develop a prominent hypoalbuminemia. This is probably due to the markedly increased permeability of the blood-joint barrier for albumin, leading to high albumin concentration in synovial fluid and inflammatory edema [22]. Wilkinson et al. found that hypoalbuminemia in RA was caused neither by failure of albumin synthesis nor by increased albumin loss. Increased vessel permeability also could not be responsible for the formation of hypoalbuminemia, because extravascular albumin pool of RA patients was decreased rather than increased. In contrast, albumin catabolism was significantly increased in RA patients and was mostly closed to the activity of the disease [23]. The studies of Ballantyne et al. and Niwa et al. further confirmed these results [24,25]. The authors suggested that the increased turnover of albumin in RA is probably due to high consumption of albumin at inflammatory sites where the synovial cells show an active metabolic state accompanied by a high demand for nitrogen and energy, and that the increased albumin production can not compensate the decreased albumin serum level in RA. The results of our present study are consistent with the findings outlined above. We could observe a lower level of albumin in the blood of the arthritic mice compared with healthy mice. Moreover, we could demonstrate an intensive accumulation of albumin in the paws affected arthritis.

Although albumin fusion technology has been widely used to extend the half-lives of hormones, cytokines, and antibody fragments, very few data are available so far about its application in targeted delivery of protein drugs to inflamed joints for RA treatment. It is well known that protein drugs fused to anti-albumin antibodies showed a dramatically prolonged half-life and selectively accumulated in the inflamed joint [27,28]. However, whether direct fusion of protein drugs to albumin has the same effect as described or not still remains to be further illustrated. This is because the proteins covalently linked to albumin may have different metabolism behaviors under *in vivo* conditions from those non-covalently bound to albumin. The results of our present study demonstrate that fusion of protein drugs to albumin is also a promising drug delivery approach for the targeted therapy of RA. After injection of radiolabeled IL-1ra and radiolabeled fusion protein to mice with or without CIA, a standard mouse model for RA, we could observe a marked gain in plasma half-life for the fusion protein. Moreover, we could demonstrate an intensive accumulation of the fusion protein in inflamed paws, but not in healthy paws. In contrast with the fusion protein, IL-1ra was rapidly removed from the blood circulation and showed no accumulation in inflamed paws. The distribution assays indicate that the fusion protein is mainly in the plasma and only a small fraction distributes in the liver, kidney, spleen and lung, similar to the distribution pattern of HSA. However, IL-1ra shows a short circulation time and high distribution rates in liver, kidney, spleen and lung. These data suggest that the unfavorable pharmacokinetic properties of IL-1ra with regard to plasma half-life and tissue specificity are substantially improved by albumin fusion. In this study, the fusion protein shows a serum half-life of bout 10 h, which is longer than that of the IL-1ra/AlbudAb (a single domain anti-albumin antibody fragment) fusion protein (4.3 h) in mice [27]. This indicates that the elimination mechanism of proteins covalently bound to albumin may be different from that of proteins non-covalently bound to albumin. The long half-life of albumin depends on the FcRn-mediated endocytosis and release pathway, whereas the non-FcRn-binding proteins are prone to be endocytosed and degraded by cell lysosome [15,16]. This suggests that proteins covalently bound to albumin are probably more resistant to degradation because they always bind to albumin under *in vivo* condition. Which one of the two approaches is more superior is worthy to be studied in the future.

CIA is a polyarthritis induced by sensitization of susceptible strains of animals with type II collagen. There are several similarities with the human RA during the disease initiation and progression, including linkage of disease to genes residing in the histocompatability locus [34], mononuclear cell infiltration, pannus development, fibrin deposition, erosion of cartilage and bone, and autoreactive T and B cells [35]. Cytokines such as TNF-α, IL-1β, and IL-6 have been shown to display potent pro-inflammatory actions that are thought to contribute to the pathogenesis of RA [36-38]. To complete the preclinical study, we investigated the efficacy of the fusion protein compared with IL-1ra in mice established CIA. A comprehensive assessment of clinical symptoms, histopathology of joints and pro-inflammatory cytokines was performed to interpret the therapeutic efficacy. Both the fusion protein and IL-1ra gave a positive effect in all these parameters. This finding presented here is consistent with the published work which reported the marked amelioration of established CIA following treatment with anti-IL-1 mAb or IL-1ra [39-42]. However, IL-1ra treatment showed only a little protection, and often the data were not significant or at the limit of significance, probably due to an insufficient dosage and a relatively long injection interval. This is in accordance with the previous study that suggested a relatively high dosage and sustained infusion of IL-1ra was needed to obtain a satisfactory efficacy [39,40]. In contrast, the fusion protein showed a much more significant efficacy in suppressing the CIA progression, with a pronounced reduction in clinical score, paw swelling, joint damage, and cytokines expression at the same dosage as that of IL-1ra. This is undoubtedly due to the extension of plasma half-life and the specific accumulation of the fusion protein in inflamed joints.

## Conclusions

Taking all the data together, albumin fused IL-1ra appears to be a suitable drug for the treatment of patients with RA. With an extended plasma half-life and selective accumulation in inflamed joints, the fusion protein promises to substitute daily infusion of high dose of IL-1ra in RA therapy with once a week dosing or longer. Additionally, genetic fusion of small protein drug to albumin is a promising approach for targeted therapy of RA.

## Methods

### Plasmid, strains and cells

Recombinant plasmid vectors pMD18T/IL-1ra and pMD18T/HSA were constructed in our laboratory. *E. coli* strain Top10 (Tiangen Biotech., Beijing, China) was used for cloning and maintaining plasmid throughout the experiments. *Pichia pastoris* host strain GS115 (his4) and the integrating plasmid vector pHBM905B were kindly provided by Professor Lixing Ma(College of Life Sciences, Hubei University, Wuhan, China) for protein expression. Human melanoma A375.S2 cells (ATCC) was stored in our laboratory and when required, cultured in RPMI-1640 medium containing 10% FBS (foetal bovine serum, Hyclone) at 37°C in a 5% CO2 incubator.

### Regents and materials

Recombinant human interleukin-1β (IL-1β) was from Peprotech (Rocky Hill, NJ, USA). Human IL-1ra and HSA were cloned and produced in *Pichia pastoris* in our laboratory using the same expression vectors and host cells as described in this study, with 6 histidines (His tag) in the carboxyl terminal. FITC-labeled anti-His tag monoclonal antibody (mAb) and HRP-conjugated anti-His tag mAb were from Santa Cruz Biotechnology (Santa Cruz, CA, USA).Restriction endonuclease, T4 DNA ligase, DNase, and RNase were from TaKaRa Biotechnology (Dalian, China). Pfu DNA polymerase was a product of BioAsia (Shanghai, China). All forward and reverse primers were purchase from BioAsia (Shanghai, China). Ni-NTA agarose was from Pharmacia Biotechnology Company (Piscataway, NJ, USA). Bovine type II collagen and Complete Freund’s adjuvant were obtained from Sigma Chemical Company (StLouis, MO, USA). The commercial ELISA kit for mouse TNF-α, IL-1β, IL-6 and albumin were from Biosource international (Camarillo, CA, USA).

### Construction of expression vectors and transformation of Pichia pastoris cells

The cDNA fragments encoding IL-1ra and HSA were obtained by PCR using plasmid vector pMD18T/IL-1ra and pMD18T/HSA as templates, respectively. The primers were as follow: P1, IL-1ra sense 5’-TTA*CGGTCCG*ATGCACACAAGAGTGAGGTT-3’; P2, IL-1ra antisense 5’-TGTGCATCTGGAGCTGGAGCTGGCTCGTCCTCCTGGAAGTAGAA-3’; P3, HSA sense 5’-GAGGACGAGCCAGCTCCAGCTCCAGATGCACACAAGAGTGAGG TT-3’; P4,HSA antisense 5’-TTA*GCGGCCGC*TTAATGATGATGATGATGATGGAATTCTAAGCCTAAGGCAGCTTGAC-3’. A *Cpo* I and a *Not* I restriction sites were introduced to the P1 and P4 respectively for cloning purpose. Primers P2 and P3 have a complementary region of 32 base pairs, containing the sequence encoding a five peptide linker PAPAP. A 6 × histidine (his tag) coding sequence was introduced into primer P4, insuring that the fusion protein could be identified by immunoblotting using anti-his tag antibody and be purified by immobilized Ni^2+^ affintity chromatography. The IL-1ra-HSA fusion gene was amplified by an overlapped PCR using a mixture of the above two PCR products as templates, with P1 and P4 as primers. The conditions for the amplification of three gene fragments were as follows: 95°C for 5 min, followed by 30 cycles of 95°C for 1 min, 55 °C for 1 min, and 72°C for 1 min. The final extension was 72 °C for 5 min. The fusion gene obtained by the PCR, encoding IL-1ra and HSA which were linked by a PAPAP peptide linker, was then digested by *Cpo*I and *Not*I and ligated into the expression vector pHBM905B digested by the same enzymes, resulting in the recombinant vector pHBM905B/IL-1ra-HSA. The recombinant vector was transformed into *E. coli* Top10 for amplification and DNA sequencing. Recombinant vector pHBM905B/IL-1ra-HSA was linearized by *Sal* I and introduced into *P*. pastoris GS115 (his4) by electroporation. Transformants were initially screened for their viability in the absence of histidine. The positive recombinants were further identified by PCR using primers P1 and P4, and were analyzed with a 0.8% agarose gel.

### Expression of fusion protein

The recombinants were inoculated in 25 ml of BMGY medium (100 mM potassium phosphate buffer, pH6.0, 1% yeast extract, 2% peptone, 4 × 10^-5^% biotin, 1% v/v glycerol) and the culture was incubated at 30°C with constant shaking to reach an A_600nm_ of 6–8. The cells were then collected by centrifugation at 3,000 g for 10 min and resuspended in 250 ml BMMY medium (BMGY with 0.5% methanol instead of 1% glycerol) in a 1-liter shake flask. The cultures were incubated for 120 h at 30°C with constant shaking, and 100% methanol was added to a final concentration of 0.5% every 12 h to maintain induction. The cultures were then centrifuged at 5,000 g for 10 min, and the supernatant was harvested and checked by 12% SDS/PAGE.

### Identification of fusion protein by immunoblotting

Immunoblotting analysis was performed according to the standard protocol. Briefly, the supernatant was sampled and separated on a 12% SDS/PAGE and transferred to a nitrocellulose membrane (Pall Gelman Sciences, Port Washington, NY, USA; 0.45 μm pore size). The membrane was blocked in 1 × PBS (pH7.4) solution with 5% nonfat dry milk at 4°C overnight, and incubated with HRP-conjugated anti-His tag mAb (1:1000) in 1 × PBS (pH7.4) at 37°C for 2 h. Finally, the membrane was washed with PBST three times and the protein was identified by visualization with diaminobenzidine (DAB) as the substrate.

### Purification of fusion protein

Identical volume of 100% saturated ammonium sulfate (pH7.0) was slowly added to the 250 ml supernatant obtained from the expression cultures, and the supernatant was stored at 4°C overnight with constant stirring, ensuring the proteins were thoroughly precipitated. The proteins were harvested by centrifugation of 10000 g at 4°C for 30 min, resusupended in 20 ml1 × PBS, and then dialyzed against 500 ml buffer of 20 mM Tris–HCl (pH8.0), 150 mM NaCl at 4°C for 24 h, changing the dialysis buffer once every 3 h to remove the residual ammonium sulfate and impurities. The protein was then purified by IMAC (immobilized-metal ion affinity chromatography). Briefly, the protein solution was loaded onto a 10 ml Ni-NTA agarose column, which was pre-equilibrated with 10-fold volume of above dialysis buffer at a flow rate of 0.5 ml/min. After loading of the mixture, the column was washed with a buffer of 20 mM Tris–HCl (pH8.0), 500 mM NaCl, 20 mM imidazole, at 1 ml/min until the absorbance reached the baseline. The target protein was then eluted with a buffer of 20 mM Tris–HCl (pH8.0), 150 mM NaCl, 500 mM imidazole at 1 ml/min, and was checked for its purity by 12% SDS/PAGE. The eluted protein solution was re-dialyzed against 100-fold volume of 1 × PBS buffer at 4°C overnight to remove the residual impurities, followed by centrifugation at 14000 g at 4°C for 30 min and passed through a 0.45 μm-pore-size filter to remove the potential aggregations and microorganisms. At last, the protein was concentrated by ultracentrifugation using centriconmicro-concentrators (Amersham Biosciences, UK), quantified by Bradford method with BSA as control and lyophilized for use.

### Analytical gel filtration

Analytical gel filtration of the fusion protein was performed on an Amersham Pharmacia Biotech HPLC system using a TSK-G3000-SW_XL_ column (7.8 mm diameter × 300 mm long; Tosoh Bioscience, Tokyo, Japan) equilibrated with degassed PBS. Portion (20 μl) of the fusion protein were injected at a concentration of 200 μg/ml. For calibration of the TSK-G3000-SW_XL_ column, the standard proteins IgG (molecular mass 150 kDa) and BSA (66 kDa) were used. The molecular mass of the fusion protein was determined according to its retention time.

### Receptor binding

Human melanoma A375.S2 cells were fixed with 4% paraformaldehyde and incubated with the fusion protein, IL-1ra, or HSA at 25°C for 2 h. After three times washing with 1 × PBS, a FITC-labeled anti-His tag monoclonal antibody (mAb) was added at a 1:500 dilution. The cells were again washed three times with 1 × PBS. Confocal microscopy was performed on an Olympus BX 50 WI microscope (Olympus, Melville, NY) coupled with a TI:Sapphire laser (Spectraphysics, Irvine, CA) and a Radiance 2000MP scanning confocal multiphoton imaging system (BioRad Laboratories, Hercules, CA). Images were taken at room temperature. Adobe Photoshop7.0 Software (Adobe, San Jose, CA) was used for subsequent image processing.

### Inhibition of IL-1β action

A375.S2 cell cultures were harvested in EDTA, washed, and resuspended to 2.5 × 10^5^ cells/ml in RPMI-1640 medium containing 10% FBS (foetal bovine serum, Hyclone). 100 μl/well of cell suspension was added to a 96-well assay plate and incubated overnight at 37°C, 5% CO_2_ in a humidified incubator. 100 μl/well of assay medium (RPMI-1640 medium containing 2% FBS) was added to another plate, and IL-1ra or fusion protein was serially diluted in the 100 μl assay medium by 2-fold from row 3 to row 12 in triplicate, followed by addition of 100 μl/well of rhIL-1β to1 ng/ml (final concentration) from row 2 to row 12. Row 1 was supplemented with 100 μl assay medium to an identical volume. 100 μl of this mixed solution was then transferred to the corresponding wells of the assay plate with cell culture. The plate was incubated for 72 h at 37°C, 5% CO_2_ in a humidified incubator. After incubation, 10 μl/well of 5 mg/ml MTT solution was added and the plate was incubated at 37°C, 5% CO_2_ for 4 h. The assay medium was decanted and to each well 50 μl of MTT lysing solution was added. A_570_ was then read and percentage inhibition was calculated with the following formula: Inhibition (%) = [[A_570_, _M_ - A_570_, _I_]/[A_570,__N_ - A_570, I_]] × 100%. Where A_570_, _I_ is the mean value of absorbance of wells with addition of only rhIL-1β; A_570_, _M_ is the mean value of absorbance of wells with addition of the mixture solution of rhIL-1β and IL-1ra or fusion protein; and A_570,__N_ is the absorbance of wells without addition of both rhIL-1β and IL-1ra or fusion protein. HSA was used as a control protein in this assay.

### Animal and arthritis model

Male DAB/1 mice, 7–8 weeks of age were obtained from Chinese Academy of Medical Science (Beijing, China). The animals were fed with standard rodent chows and water, and the health status was monitored everyday. All animal experiments were designed and conducted according to the recommendations of *Guidelines for Experimental Animal Administration* approved by Institutional Animal Care and Use Committee at the Hubei Experimental Animal Regulation Board (Ezhengfa/1993/79). Collagen-induced arthritis (CIA) was induced in DAB/1 mice as described previously, by multiple intradermal injections at the base of the tail and into three or five other sites on the back, of 250 μg of bovine CII (type II collagen) in 125 μl of 0.1 M acetic acid emulsified in an equal volume of Complete Freund’s adjuvant containing 2 mg dry weight of Mycobacterium tuberculosis/ml. Mice were challenged again with the same dose of preparation three weeks later by an intraperitoneal injection. Disease developed about 11 days after the second immunization. The arthritis model in mice characterized by erythema and swelling of the paws in one or more limbs.

### Albumin measurement

5 mice with CIA and 5 healthy mice were sacrificed at the time when the arthritis score arrived at a mean value of above 10 per mouse. The blood samples were drawn and serum albumin was analyzed using a commercial quantitative ELISA kit according to the manufacturer’s instruction. Data were expressed as g albumin/L serum. Albumin levels in the paws of these mice were measured by quantification of albumin in the joint extractions using a commercial quantitative ELISA kit. Joint extractions were prepared in the same way as that used for joint cytokines measurement described below. Data were expressed as mg albumin/g tissue.

### Pharmacokinetics and distribution of fusion protein

To qualify the circulation times and distribution of fusion protein in health and inflamed joints, as well as in the liver, kidney, spleen, and lung at different time points, 55 mice with CIA and 55 healthy control mice were randomly divided into eight groups, and received an intravenous injection of 7.4 MBq of the radiolabeled fusion protein IL-1ra-HSA (^131^I-IL-1ra-HSA), dissolved in 50 μl PBS (pH7.4). Five mice with CIA and five healthy mice were sacrificed at the time points of 10 min, 30 min, 1, 3, 8, 13, 24, 48, 72, 96, and 120 h after tracer injection. All paws, livers, spleens, lungs and kidneys were prepared and weighed, and blood samples were drawn. Radioactivity of blood and other tissues were measured using a gamma-scintillation counter (Berthold, Wildbad, Germany). The uptakes of the fusion protein by the paws and other tissues were calculated as a percentage of the initially injected radioactivity and the half-life in circulation was obtained from the analysis of the plot of percentage of radioactivity in the blood samples over time with a one-phasic exponential function (GraphPad software). The paws of mice sacrificed after 48 h were detected for the selective accumulation of the radiolabeled fusion protein in inflamed and normal joints by autoradiography using a phosphorimager screen (Fujifilm, Dielsdorf, Switzerland).

To compare the pharmacokinetics of the fusion protein with that of IL-1ra, 7.4 MBq of radiolabeled IL-1ra (^131^I- IL-1ra), dissolved in 50 μl PBS (pH7.4), was intravenously injected into 40 mice with CIA and 40 health control mice, randomly divided into eight groups. At the time points of 10 min, 30 min, 1, 3, 8, 13, 24, and 48 h, five mice with CIA and five health mice were sacrificed and all paws, livers, lungs, spleens, kidneys and blood samples were prepared. The uptakes of IL-1ra by paws and other organs, and the half-life in circulation were calculated as mentioned above.

### Treatment of mice CIA with fusion protein

On the 11th day after the second immunization, mice were subdivided into five groups and receive the treatment as follows: (1) normal control (n = 5); (2); CIA plus saline (n = 5); (3) CIA plus HSA (n = 5); (4) CIA plus IL-1ra (n = 5); (5) CIA plus fusion protein (n = 5). HSA, IL-1ra, and fusion protein were each dissolved in physiological saline (0.9% NaCl) to a concentration of 1 mg/ml, and administered every other day by intraperitoneal injection of 10 mg/kg body weight. The CIA control mice receive intraperitoneal injection of physiological saline, coinciding with the injection time of other groups. The treatment continued for three weeks.

### Arthritis assessment

The severity of the arthritis was monitored at 2-day intervals using two disease indices (clinical score and paw swelling). The clinical score for each limb was graded from 0 to 4 as follows: 0 = normal; 1 = erythema; 2 = erythema plus slight swelling; 3 = pronounced edematous swelling; 4 = joint rigidity or deformation. Each limb was graded, giving a maximal score of 16 per mouse. Clinical severity was also assessed by paw swelling, obtained by measuring the thickness of each paw with a dial-gauge caliper. The result was expressed as percentage increment in paw thickness relative to the paw thickness before the onset of arthritis.

### Histological analysis

Mice were killed at the end of the 21 days of therapy by diethyl ether narcosis; hind limbs of each animal from different experimental groups were removed and fixed in 10% formalin buffer. The limbs were decalcified in 5% (v/v) nitric acid, processed for paraffin embedding, sectioned at 5 μm thickness for the ankle joints, and subsequently stained with haematoxylin and eosin for examination under an optical microscope. All ankle joints were scored in a blind fashion for synovitis, bone erosions, cartilage damage and fibrous inflammatory hyperplasia using a predefined scoring system [43]. Briefly, the severity of each kind of the four pathological changes was assessed using a semi-quantification method and classified as normal, mild, moderate or severe on the basis of the following criteria: 0 = normal, no change; 1 = mild, minimal pathological change limited to discrete foci; 2 = moderate, pathological change present but normal joint architecture intact; 3 = severe, severe pathological change and joint architecture disrupted. The histological score for each pathological change was calculated by multiplying its score by its weight (synovitis, bone erosions, and cartilage damage, ×1; fibrous inflammatory hyperplasia, ×3), on the basis of the change’s importance in the pathology of mice CIA. The total histological score for each joint was obtained by addition of the histological score given for each of the four pathological changes, giving a maximal value 18 for a joint.

### Joints cytokines measurement

Excised ankle and paw joints (including synovium, adjacent tissues and bones) of individual mouse from each experiment group were immediately frozen in liquid nitrogen and pulverized using a mortar and pester. Tissue was transferred to 15 ml tubes, placed on ice and resuspended in 1 ml 1 × PBS/200 mg of tissue (containing protease inhibitor Phenylmethanesulfonyl fluoride) and homogenized using a Biospec Tissue-Tearor (Biospec Inc, USA) for 30 s. The homogenates were centrifuged at 1000 g for 15 min at 4°C. Supernatants were transferred to 1.5 ml eppendorf tubes, centrifuged at 15000 g for 10 min and collected for cytokines analysis using the commercial quantitative ELISA kits for mouse TNF-α, IL-1β, and IL-6 according to the manufacturer’s instruction. Data were expressed as pg cytokine/g tissue.

### Statistical analysis

Data were expressed as mean ± SEM. The difference between the means of two groups were determined with Student’s *t* test and was considered significant at p < 0.05.

## Abbreviations

IL-1ra: Interleukin-1 receptor antagonist; RA: Rheumatoid Arthritis; CIA: Collagen-induced Arthritis; HSA: Human Serum Albumin; HRP: Horseradish Peroxidase; FITC: Fluoresceinisothiocyanate; IL-1β: Interleukin-1β; IL-6: Interleukin-6; TNF-α: Tumor Necrosis Factor; IMAC: Immobilized-metal Affinity Chromatography; MTT: 3-(4,5-dimethylthiazol-2-yl)-2,5-diphenyl-2 H-tetrazolium bromide; PBST: Phosphate Buffered Saline containing 0.5% Tween 20; Ni-NTA: Ni^2+^-nitrilotriacetic acid; ELISA: Enzyme-linked Immunosorbent Assay; PCR: Polymerase Chain reaction.

## Competing interests

All authors of the manuscript declare that they have no financial and non-financial competing interests with any organization as well as personal, religious, ideological, academic, intellectual, commercial or any other situations.

## Authors’ contributions

ML conceived the idea, initiated and supervised the study, and wrote the mauscript. YH carried out the protein expression, purification, and the main experiments including the pharmacokinetics and pharmacodynamic assay. LH performed the molecular cloning and construction of the expression vector. XH carried out the in vitro bioactivity assays including immunofluorescence and neutralization of IL-1β action. GL fed the animal, established the CIA model, and treated the animal with fusion protein. DL and XY participated in data interpretation and revision of the manuscript. All authors read and approved the final manuscript.
